# Blood pressure control amongst patients living with hypertension presenting to an urban district hospital outpatient clinic in Kwazulu-Natal

**DOI:** 10.4102/phcfm.v6i1.572

**Published:** 2014-07-28

**Authors:** Folafolu A. Adebolu, Mergan Naidoo

**Affiliations:** 1 Department of Family Medicine, University of Kwazulu-Natal, South Africa

## Abstract

**Background:**

The prevalence of hypertension in South Africa has been estimated to be 20% of the adult population with over six million people being affected. Poor adherence to treatment plans lead to inadequate blood pressure control and high morbidity. Many studies have looked at factors contributing to poor blood pressure control in South Africa but few studies actually focus on district hospitals in Kwazulu-Natal in particular, despite the fact that the province has the most heterogeneous population in South Africa.

**Method:**

The study was a descriptive cross-sectional study conducted at the chronic outpatient clinic of an urban district hospital involving 370 participants aged 18–90 years.

**Result:**

The study showed poorly controlled blood pressure in 58% of the participants. Only 35% knew their blood pressure results on the day of interview and 19.2% were aware of their target blood pressure. Good adherence was self-reported by 95% of the participants, whist 51.4% reported significant side-effects to medication.

**Conclusion:**

The majority of patients had poor knowledge about blood pressure and little awareness of their blood pressure reading. These may be precursors to poor blood pressure control and this needs further investigation. A high level of self-reported adherence to medication did not translate into effective blood pressure control. A significant number reported medication side-effects which may have contributed to the poor blood pressure control. The high adherence rate may therefore have been over reported. An objective way to measure adherence will be necessary for future research.

## Abstrait

**Contrôle de la tension artérielle des patients souffrant d'hypertension qui se rendent aux consultations externes d'un hôpital urbain de district au Kwazulu-Natal**.

**Contexte:**

La prévalence de l'hypertension en Afrique du Sud est estimée à 20% de la population adulte, soit plus de six millions de personnes. La non-observation du plan de traitement mène au contrôle insuffisant de la tension artérielle et à un taux élevé de décès. De nombreuses études ont examiné les facteurs contribuant au contrôle insuffisant de la tension artérielle en Afrique du Sud, mais peu d’études se sont réellement penchées sur les hôpitaux de district du Kwazulu-Natal en particulier, en dépit du fait que la province a la population la plus hétérogène d'Afrique du Sud.

**Méthode:**

L’étude descriptive et transversale a été menée à la clinique de consultations externes pour malades chroniques d'un hôpital urbain de district, parmi 370 participants âgés de 18 à 90 ans.

**Résultat:**

L’étude a révélé une tension artérielle mal contrôlée chez 58% des participants. Seuls 35% d'entre eux savaient quelle était leur tension artérielle le jour de l'entrevue et 19.2% savaient la tension artérielle visée. 95% des patients ont déclarés qu'ils suivaient le traitement, tandis que 51.4% ont déclarés que le médicament avait des effets secondaires significatifs.

**Conclusion:**

La majorité des patients avait peu de connaissances sur la tension artérielle et ils n’étaient pas au courant de leur taux de tension. Cela peut être l'indication d'un mauvais contrôle de la tension artérielle et doit faire l'objet de recherches supplémentaires. Le taux élevé d'adhésion auto-déclarée au traitement ne se traduit pas par un contrôle efficace de la tension artérielle. Un nombre significatif de patients a déclaré avoir des effets secondaires au traitement, ce qui peut avoir contribué au mauvais contrôle de la tension. Ainsi, le taux élevé d'adhérence peut avoir été sur-déclaré. Pour les recherches futures il faudra trouver une manière plus objective de mesurer l'adhésion aux médicaments.

## Introduction

Hypertension is a major worldwide health challenge and a key risk factor responsible for adverse cardiovascular events. Although, largely preventable and modifiable, uncontrolled hypertension has been reported to negatively affect the global population's health, causing cerebrovascular accidents and cardiac diseases.^1^

Hypertension ‘accounts for greater than 5.8% of the total deaths, 11.9% of the years of life lost, and 1.4% of the disability-adjusted life years worldwide’.^2^ It has a major influence in the development of cardiac and vascular diseases. Despite being a highly treatable condition, studies have shown that hypertension remains poorly controlled.^3^ In addition, the diagnosis, management, and control of hypertension pose a great challenge for physicians and researchers.^4^

Hypertension affects approximately one billion individuals worldwide and fifty million individuals in the United States of America.^5^ A number of clinical trials have shown that optimal blood pressure (BP) control can be achieved in most hypertensive patients using multiple antihypertensive medications.^6^ A retrospective study conducted in community-based settings in the USA indicated that 70% of the population had blood pressure levels that were optimally controlled. This was similar to the National Health and Nutrition Examination Survey (NHANNES) study done between 2007 and 2008, which indicated that 69% of patients in this cohort had controlled blood pressure. This study showed that optimally controlled patients living with hypertension were significantly more likely to be younger and have less co-morbidity.^7^ A study which looked at uncontrolled hypertension in the United States concluded that the majority of cases were due to systolic hypertension. This report reaffirms the need to assess the extent to which patients are aware of their systolic blood pressure as systolic blood pressure awareness and education is an important component of the interventional programme that is used in the control of hypertension.^7, 8^

Hypertension control has been positively linked to adequate knowledge about blood pressure goals. A recent study done on 7649 participants across Europe showed that poor blood pressure control bears a strong correlation to lack of knowledge of target blood pressure. The study found that 50.4% of participants had controlled blood pressure and 49.4% had knowledge of their target blood pressure.^9^ Numerous studies have concluded that poor medication adherence, lack of patient awareness about their target blood pressure levels, physician inertia as well as the presence of drug side-effects should be considered as possible causes of inadequately controlled blood pressure.^3^

Adequate assessment and treatment of hypertension has always been a major challenge for public health care workers.^5^ An article in Time magazine in 2004 stated that ‘with sub-Saharan Africa's population of 650 million and increasing longevity and westernization, hypertension has now changed from a relatively rare condition to a major problem. This observation necessitated the African Union to regard hypertension as one of the continent's greatest challenges after AIDS’.^10^ The prevalence of hypertension in rural studies undertaken in sub-Saharan Africa in the 1970s, 1980s and 1990s using the previous definition of blood pressure of 160/95 mmHg found hypertension to be as low as 4.1% in Ghana, 7.0% in Lesotho, 5.9% in Nigeria, and 7.37% in rural Zululand in South Africa.^11^

Estimates based on the 1998 demographic survey put the prevalence of hypertension at 11% and 14% of South African males and females respectively. This implies that there were six million hypertensive persons in South Africa.^12^ This number is likely to be even higher in 2012 because of population growth and growing urbanisation.^13^ In the year 2000, 47 000 deaths in South Africa were attributed to high blood pressure, and elevated blood pressure was the second leading contributory cause of death.^14^

A study amongst Durban adults reporting hypertension trends using the WHO criteria of 160/90 mmHg found hypertension to be more common in urban black South Africans of Zulu origin (25%), moderate in white South Africans (17%), and Indian populations (14%), and the lowest in rural black South Africans.^15^ The 2011 South African Hypertension Guidelines recommend that the target blood pressure for patients on hypertensive therapy should be below 140/90.^16^ In patients with major cardiovascular risks such as those with diabetes mellitus, associated clinical conditions or established target organ damage, the optimal blood pressure should be 130/80 mmHg.^16^

In well conducted clinical trials, antihypertensive drugs either as mono-therapy or combination therapy have been shown to effectively control blood pressure and reduce cardiovascular morbidity and mortality.^17^ However, outside of clinical trials, control of hypertension remains disappointing with an estimated 30% of patients on treatment being effectively controlled.^16, 18^

Despite the availability of multiple effective antihypertensive drugs, control of hypertension remains poor.^5, 19^ In both high and low income countries, less than 27% and 10% respectively of hypertensive patients have achieved their target blood pressure.^5, 19^

Many reasons have been ascribed for the poor control of hypertension worldwide. Patient knowledge of hypertension also plays an important role in the ability to achieve successful control.^17^ In addition, poor treatment adherence, inappropriate prescriptions, therapeutic inertia and unhealthy lifestyle contribute to poor control.^3, 20, 21^ The 2011 South African Hypertension Guidelines emphasised poor adherence to medication as one of the main causes of uncontrolled blood pressure and was thought to be due to treatment factors, patient factors and illness related factors.^16^ Other factors identified as contributors to uncontrolled hypertension include sex, age, and race.^8^ In South Africa, white and Asian individuals have been noted to have better blood pressure control compared to their black counterparts.^12^ Black individuals also develop hypertension earlier in life, have more severe hypertension related complications and respond differently to antihypertensive medication.^22^

In the first South African Demographic and Health Survey (1998), awareness, treatment status, and control varied substantially between men and women, as well as between different ethnic groups.^12^ Women were substantially more likely to be aware of their hypertension than men, 51% and 26% respectively. The population survey also found that 36% of women and 21% of men had hypertension but were not on treatment. Amongst those on treatment, only 18% were controlled. With respect to ethnic differences, black South Africans had the lowest rates of awareness, treatment rates and control of hypertension.^12^

There is little published data on hypertension control amongst patients attending district hospitals in the KwaZulu-Natal Province, South Africa. The aim of this research was to describe the pattern of blood pressure control amongst patients attending a busy outpatient clinic in an urban district hospital. The specific objectives were to describe the demographic and clinical characteristics, knowledge of hypertension targets, degree of adherence to medication and knowledge of side-effects to antihypertensive medications. Approval for the study was granted by the Bioethics Research Committee of the University of KwaZulu-Natal (UKZN), the postgraduate education committee, hospital management and the KwaZulu-Natal Department of Health. Written consent was obtained from all study participants.

## Methods

This was a descriptive, cross-sectional study done at the chronic outpatient clinic at a busy urban public health district hospital. An average of 1375 hypertensive patients was seen monthly at the outpatient clinic in 2011. A sample size of 370 was selected as being appropriate for an observational descriptive study as confirmed with a biostatistician. Hypertensive patients visiting the outpatient clinic were systematically sampled by selecting every third patient that met the inclusion criteria over a three month period commencing October 2012. Sampling was stopped when the sample size was attained. The inclusion criteria were all patients living with hypertension older than 18 years attending the chronic clinic, and on treatment for hypertension for at least one year. Excluded from the study were pregnant women and cognitively impaired patients. Patients’ clinical records were reviewed and an exit structured interview was conducted with each participant by the principal investigator after the participant had consulted the hospital medical officer. The study tool captured demographic data, anthropometric data and, clinical information from the clinical record. Patients were asked specific questions about their hypertension, co-morbid conditions, and habits. Blood pressure readings from the last two clinic visits plus current blood pressure readings were captured. Poor control was defined as blood pressures greater than 140 mmHg systolic or 90 mmHg diastolic. Patients who had two of the three readings elevated were considered to be poorly controlled. Data was captured and analysed using SPSS Statistics for Window Version 21.0. (Armonk, NY: IBM Corp 2012). Results were summarised by frequencies and percentages (categorical variables) and mean, median, standard deviation and percentiles (numerical variables) as appropriate. Wilcoxon signed rank test was used in analysing the variations and trends between the categorical data.

## Results

Forty-nine percent of the participants were black South Africans, 25.4% were Indians, 17.8% were Mixed race and 7.6% were white South Africans with their age groups being presented in Table 1. Approximately 62.3% of the participants had secondary and tertiary education, 59.1% were pensioners or patients collecting a social grant. Sixty percent of the participants were either overweight or obese.

**TABLE 1:** Demographic details (*n* = 370).

Variables	Number	% of uncontrolled BP in each group
**Age**		
18–37	19	57.8
38–57	132	51.5
58–77	187	63.6
78–97	32	56.2
**Sex**		
Male	111	56.7
Female	259	59.0
**Marital status**		
Single	111	62.1
Married	160	60.0
Divorced	30	60.0
Widowed	69	59.4
**Race**		
Black South African	182	65.9
White South African	28	35.7
Mixed Race	66	62.1
Indian	94	47.8
**Education level**		
No education	44	63.6
Primary	95	58.9
Secondary	203	56.6
Tertiary	28	57.1
**BMI (Kg/m** ^2^ **)**		
< 18	34	2.94
18–24	113	61.0
25–29	89	61.7
> 30	134	67.9

BP, blood pressure; BMI, body-mass index.

The study showed uncontrolled blood pressure (BP) in 58% of the participants during the preceding three months. Other determinants of blood pressure control are shown below in Table 2. Eighty percent had other co-morbid diseases such as diabetes mellitus, asthma or arthritis and 60% were either obese or overweight. Approximately 81% did not know what their target blood pressure should be, and 65% could not recall what their blood pressure for the current visit was. Most participants claimed that they were not told what their blood pressure was by the health care providers managing their hypertension. Eighty-eight percent of participants claimed a 95% adherence to treatment. This information was not verified. Fifty-one percent of the participants reported significant side-effects ranging from cough, dizziness, skin rashes and urinary frequency, but almost 60% of these did not know if the drug treatment was causing these side-effects. More than 70% of the study participants indicated that they practiced at least one form of exercise to decrease their weight. Only 48% of the hypertensive patients could describe what a DASH diet was and these participants claimed to adhere to it. Sixty-six percent of the participants claimed to be psychologically stressed.

**TABLE 2:** Determinants of blood pressure control.

Variables	Number	%
**Blood pressure**		
Controlled	155	41.9
Not controlled	215	58.1
**No of antihypertensive medication**		
One	13	3.5
Two	70	18.9
Three	108	29.2
> Three	179	48.4
**Naming of antihypertensive medications**		
Adequate knowledge	77	20.8
Partial knowledge	88	23.7
No knowledge	205	55.5
**Adherence**		
> 95%	327	88.4
< 95%	43	11.6
**Side effects to medication**		
Yes	190	51.4
No	180	48.6
**Knowledge of target blood pressure**		
Adequate knowledge	71	19.2
No knowledge	299	80.8
**Presence of co-morbidity**		
Yes	297	80.3
No	73	19.7
**Investigations**		
No investigation in clinical record	171	46.2
Partially investigated	120	32.4
Investigated according to guidelines	79	21.4
**Patients view about their weight**		
Normal	237	64.1
Obese	120	32.4
Underweight	13	3.5
**Knowledge about DASH diet**		
Adequate knowledge	178	48.1
No knowledge	192	51.9
**Presence of stress**		
Stress present	245	66.2
Stress absent	125	33.8
**Knowledge of BP reading at this consultation**		
Adequate knowledge	127	34.3
No knowledge	243	65.7

BP, blood pressure.

The associations between the various factors contributing towards blood pressure control are shown in Table 3 A and B below. There was no statistically significant difference between blood pressure control, and adherence (*p* = 0.514). There was no statistically significantly association between blood pressure control and increase in number of antihypertensive medications used (*p* = 0.527).There was a statistically significant association between side- effects to medication and blood pressure control (*p* = 0.004). There was a statistically significant association between BP control and ethnic group (*p* = 0.004).

**TABLE 3a:** Crosstabs of blood pressure control versus determinant factors.

BP versus adherence	Adherence > 95%	Adherence < 95%	*p*-value
BP controlled (%)	36.5	5.4	0.514
BP not controlled (%)	51.9	6.2	

BP, blood pressure.

**TABLE 3b:** Crosstabs of blood pressure control versus determinant factors.

BP versus associated co-morbidity	Co-morbidity present	Co-morbidity absent	*p*-value
BP controlled (%)	31.6	9.4	0.068
BP not controlled (%)	48.6	10.3	

BP, blood pressure.

**TABLE 3c:** Crosstabs of blood pressure control versus determinant factors.

BP versus presence of side effect	Side effect of drug reported	Side effect of drug not reported	*p*-value
BP controlled (%)	17.6	24.9	0.004
BP not controlled (%)	33.8	23.8	

BP, blood pressure.

**TABLE 3d:** Crosstabs of blood pressure control versus determinant factors.

BP control versus knowledge of target BP	Good knowledge of target BP	No knowledge of target BP	*p*-value
BP controlled (%)	17.5	82.5	0.585
BP not controlled (%)	29.9	79.1	

BP, blood pressure.

**TABLE 3e:** Crosstabs of blood pressure control versus determinant factors.

BP versus determinant	Medications prescribed				*p*-value

	Number of medicine	Number of medicine	Number of medicine	Number of medicine
BP versus number of medicine	1	2	3	> 3	0.527
BP controlled (%)	1.6	9.1	12.4	18.6	
BP not controlled (%)	1.9	9.7	16.8	29.7	

BP, blood pressure.

**TABLE 3f:** Crosstabs of blood pressure control versus determinant factors.

BP versus determinant	Black people	White people	Mixed race	Asian	*p*-value
BP controlled (%)	40.3	11.0	16.9	31.8	0.004
BP not controlled (%)	55.6	4.6	19.0	20.8	

BP, blood pressure.

## Discussion

The study showed a blood pressure control rate of 41.9% amongst the patients attending a district hospital in Kwazulu-Natal. The control rate was slightly higher than the average rate of 40% that was previously reported in South Africa but consistent with a global control rate of 19% – 54%.^23^ However, this degree of control is too low considering the complications of hypertension. Poor blood pressure control translates into increased cardiovascular morbidity and mortality such as ischemic heart diseases which affects the burden placed on health care services in South Africa.

The demographics of the study group presented in Table 1 showed that the predominant racial group in this study was black South African people and the predominant gender was female. The cross tabulation for BP control versus race showed a significant relationship (*p* = 0.004.) These further affirm findings of earlier studies which showed highest reported cases to be amongst urban black South Africans and females when viewed along ethnic and gender lines.^12, 15^

It has been argued that greater medication adherence and better blood pressure control improves with patients’ knowledge of hypertension.^24^ This study showed that only 19.2% of the participants had knowledge of their target blood pressure and the cross tabulation for the blood pressure control vs. knowledge of target blood pressure did not show a statistically significant relationship (*p* = 0.585).

The self-reported adherence rate of 88.4% does not correlate with the degree of control noted (42%). A high adherence should translate into a well-controlled blood pressure. The cross tabulation between BP control and side-effects indicated that there is a statistically significant association between side- effects reported and BP control. This would suggest that patients who experienced side- effects to drugs may have over reported their adherence rates. This over reporting of adherence is consistent with findings from other studies.^25, 26^

This study showed that 51% of the participants reported side-effects to antihypertensive medications but 60% of these participants could not identify which drugs caused the side-effects. It has been reported that 35% of hypertensive patients will stop taking their drugs in the first six months if they experience a side-effect. Another reported reason for stopping medication was patient dissatisfaction with treatment.^27^ The significantly higher proportion of side-effects noted in this study was associated with multiple antihypertensive medications and this was statistically significant (*p* = 0.042).

This study showed that 70% of the participants were using more than three medications, which is of concern as they constituted the largest percentage of uncontrolled hypertensive patients. These participants would be classified as having resistant hypertension and in South Africa should have been managed at a regional level hospital. The study also showed that 80.3% of the participants had other co-morbid diseases necessitating poly-pharmacy, which increased the possibility of adverse drug-drug interactions.

## Implications of the research

The findings of this study support previous studies which demonstrated that only about 40% of hypertensive patients attain blood pressure control in South Africa. A plan to improve the control of blood pressure from the current 42% to greater than 80% is urgently required. The poor knowledge of blood pressure control and hypertensive targets amongst this cohort of participants should be addressed by clinicians through improved patient education. The high number of reported side-effects to anti-hypertensive medication should alert physicians that prescription practices in hypertensive patients may need reviewing. In addition, quality assurance teams at district level should be encouraged to do regular clinical audits of the management of hypertensive patients and monitor outcomes. Regular monitoring and evaluation of hypertension should be part of routine clinical practice and poorly controlled patients with resistant hypertension must be referred to higher levels of care. This study supports the development and implementation of educational programmes that improve the knowledge of hypertension in patients.

## Recommendations

Educational intervention aimed at counselling patients about the cardiovascular implications should be instituted. Pharmacists, nurses and doctors managing hypertensive patients should aim to address the gaps in the management of hypertension identified by this study. There is need for future research on patients’ adherence rates using a more objective approach than self-reporting.

## Limitations

It is worth mentioning that a degree of bias may exist in this study. Adherence rates may have been over reported as the investigator is a doctor known to patients at the clinic. The predominant age group are elderly with multiple co-morbidities, which might have made hypertension control more difficult. The generalisation of these results should be limited to urban district hospitals and communities with heterogeneous ethnic populations.

## References

[1] Williams B. The year in hypertension. J Am Coll Cardiol. 2009;55(1):65–73. http://dx.doi.org/10.1016/j.jacc.2009.08.037 20117366 10.1016/j.jacc.2009.08.037

[2] Machado M, Bajcar J, Guzzo GC, Einarson TR. Sensitivity of patient outcomes to pharmacist interventions. Part II: Systematic review and meta-analysis in hypertension management. The Annals of Pharmacotherapy. 2007;41(11):1770–1781. http://dx.doi.org/10.1345/aph.1K311 17925496 10.1345/aph.1K311

[3] Morgado M, Rolo S, Macedo AF, Pereira L, Castelo-Branco M. Predictors of uncontrolled hypertension and antihypertensive medication nonadherence. Journal of Cardiovascular Disease Research. 2010;1(4):196–202. http://dx.doi.org/10.4103/0975-3583.74263 21264184 10.4103/0975-3583.74263 PMC3023897

[4] Kannel WB. Blood pressure as a cardiovascular risk factor: Prevention and treatment JAMA: The Journal of the American Medical Association. 1996;275(20):1571–1576. http://dx.doi.org/10.1001/jama.1996.03530440051036 8622248

[5] Chobanian AV, Bakris GL, Black HR, Cushman WC, Green LA, Izzo JL, et al. Seventh Report of the Joint National Committee on Prevention, Detection, Evaluation, and Treatment of High Blood Pressure. Hypertension. 2003;42(6):1206–1252. http://dx.doi.org/10.1161/01.HYP.0000107251.49515.c2 14656957 10.1161/01.HYP.0000107251.49515.c2

[6] Black HR, Elliott WJ, Neaton JD, Grandits G, Grambsch P, Grimm RH, et al. Baseline characteristics and early blood pressure control in the CONVINCE trial. Hypertension. 2001;37(1):12–18. http://dx.doi.org/10.1161/01.HYP.37.1.12 11208750 10.1161/01.hyp.37.1.12

[7] Knight E, Bohn R, Wang P, Glynn R, Mogun H, Avorn J. Predictors of uncontrolled hypertension in ambulatory patient. Hypertension. 2001;38(4):809–814. http://dx.doi.org/10.1161/hy0901.091681 11641291 10.1161/hy0901.091681

[8] Hyman DJ, Pavlik VN. Characteristics of patients with uncontrolled hypertension in the United States. New England Journal of Medicine. 2001;345(7):479–486. http://dx.doi.org/10.1056/NEJMoa010273 11519501 10.1056/NEJMoa010273

[9] Pruggera C, Keila U, Wellmanna Jr, Bacquerb DD, Backerb GD, Ambrosioc GB, et al. Blood pressure control and knowledge of target blood pressure in coronary patients across Europe: Results from the EUROASPIRE III survey. Journal of Hypertension 2011;29(8):1641–1648. http://dx.doi.org/10.1097/HJH.0b013e328348efa7 21720270 10.1097/HJH.0b013e328348efa7

[10] Kluger J. Blowing a gasket. Time. 2004;December 164(23):34–40. 15620060

[11] Seedat Y. Hypertension in developing nations in sub-Saharan Africa. Journal of Human Hypertension. 2000;14(10–11):739–747. http://dx.doi.org/10.1038/sj.jhh.1001059 11095164 10.1038/sj.jhh.1001059

[12] Steyn K, Gaziano TA, Bradshaw D, Laubscher R, Fourie J. Hypertension in South African adults: Results from the Demographic and Health Survey, 1998. Journal of Hypertension. 2001;19(10):1717–1725. http://dx.doi.org/10.1097/00004872-200110000-00004 11593090 10.1097/00004872-200110000-00004

[13] Twagirumukiza M, Van Bortel LM. Management of hypertension at the community level in sub-Saharan Africa (SSA): towards a rational use of available resources. J Hum Hypertens. 2011;25(1):47–56. http://dx.doi.org/10.1038/jhh.2010.32 20336148 10.1038/jhh.2010.32

[14] Norman R, Gaziano T, Laubscher R, Steyn K, Bradshaw D, Collaboration SACRA Estimating the burden of disease attributable to high blood pressure in South Africa in 2000. South African Medical Journal. 2007;97(8):692–698. 17952226

[15] Seedat YK. Race, environment and blood pressure: The South African experience. Journal of Hypertension. 1983;1(1):7–12. http://dx.doi.org/10.1097/00004872-198306000-00003 6681027

[16] Seedat YK, Rayner BL South African hypertension guideline 2011. South African Medical Journal. 2012;102(1):60–83. 22273141

[17] Ragot S, Sosner P, Bouche G, Guillemain J, Herpin D. Appraisal of the knowledge of hypertensive patients and assessment of the role of the pharmacists in the management of hypertension: Results of a regional survey. J Hum Hypertens. 2005;19(7):577–584. http://dx.doi.org/10.1038/sj.jhh.1001859 15830000 10.1038/sj.jhh.1001859

[18] Ly N, McCaig L. National Hospital Ambulatory Medical Care Survey: 2000 outpatient department summary Maryland, USA: 2000. 12661587

[19] Pereira M, Lunet N, Azevedo A, Barros H. Differences in prevalence, awareness, treatment and control of hypertension between developing and developed countries. Journal of Hypertension. 2009;27(5):963–975 http://dx.doi.org/10.1097/HJH.0b013e3283282f65 19402221 10.1097/hjh.0b013e3283282f65

[20] Ong KL, Cheung BMY, Man YB, Lau CP, Lam KSL. Prevalence, awareness, treatment, and control of hypertension among United States adults 1999–2004. Hypertension. 2007;49(1):69–75. http://dx.doi.org/10.1161/01.HYP.0000252676.46043.18 17159087 10.1161/01.HYP.0000252676.46043.18

[21] Rodríguez Pérez MC, Cabrera de León A, Morales Torres RM, Domínguez Coello S, Alemán Sánchez JJ, Brito Díaz B, et al. Factors associated with knowledge and control of arterial hypertension in the Canary Islands. Revista Española de Cardiología. 2012;65(3):234–240. http://dx.doi.org/10.1016/j.recesp.2011.09.021 22209706 10.1016/j.recesp.2011.09.021

[22] Opie LH, Seedat YK. Hypertension in sub-Saharan African populations. Circulation. 2005;112(23):3562–3568. http://dx.doi.org/10.1161/CIRCULATIONAHA.105.539569 16330697 10.1161/CIRCULATIONAHA.105.539569

[23] Rayner B. Combination therapy in hypertension. SA Pharmaceutical Journal. 2007;74(6):30–34.

[24] Kim E-Y, Han H-R, Jeong S, Kim KB, Park H, Kang E, et al. Does knowledge matter?: Intentional medication nonadherence among middle-aged Korean Americans with high blood pressure. Journal of Cardiovascular Nursing. 2007;22(5):397–404 http://dx.doi.org/10.1097/01.JCN.0000287038.23186.bd 17724422 10.1097/01.JCN.0000287038.23186.bd

[25] Voils CI, Hoyle RH, Thorpe CT, Maciejewski ML, Yancy Jr WS. Improving the measurement of self-reported medication nonadherence. Journal of Clinical Epidemiology. 2011;64(3):250–254. http://dx.doi.org/10.1016/j.jclinepi.2010.07.014 21194887 10.1016/j.jclinepi.2010.07.014 PMC3634915

[26] Svarstad BL, Chewning BA, Sleath BL, Claesson C. The brief medication questionnaire: A tool for screening patient adherence and barriers to adherence. Patient Education and Counseling. 1999;37(2):113–124. http://dx.doi.org/10.1016/S0738-3991(98)00107-4 14528539 10.1016/s0738-3991(98)00107-4

[27] Trinder YA. Common and less common adverse effects of antihypertensives: A general practitioner's perspective. South African Family Practice. 2012 54(2):31–32.

